# Saitohin Q7R polymorphism is associated with late‐onset Alzheimer's disease susceptibility among caucasian populations: a meta‐analysis

**DOI:** 10.1111/jcmm.13079

**Published:** 2017-02-17

**Authors:** Rong Huang, Sai Tian, Rongrong Cai, Jie Sun, Wenqing Xia, Xue Dong, Yanjue Shen, Shaohua Wang

**Affiliations:** ^1^ Department of Endocrinology Affiliated Zhongda Hospital of Southeast University Nanjing China

**Keywords:** Saitohin, Alzheimer's disease, polymorphism, meta‐analysis

## Abstract

Saitohin (STH) Q7R polymorphism has been reported to influence the individual's susceptibility to Alzheimer's disease (AD); however, conclusions remain controversial. Therefore, we performed this meta‐analysis to explore the association between STH Q7R polymorphism and AD risk. Systematic literature searches were performed in the PubMed, Embase, Cochrane Library and Web of Science for studies published before 31 August 2016. Pooled odds ratios (ORs) and 95% confidence intervals (CIs) were calculated to assess the strength of the association using a fixed‐ or random‐effects model. Subgroup analyses, Galbraith plot and sensitivity analyses were also performed. All statistical analyses were performed with STATA Version 12.0. A total of 19 case–control studies from 17 publications with 4387 cases and 3972 controls were included in our meta‐analysis. The results showed that the Q7R polymorphism was significantly associated with an increased risk of AD in a recessive model (RR *versus* QQ+QR, OR = 1.27, 95% CI = 1.01–1.60, *P* = 0.040). After excluding the four studies not carried out in caucasians, the overall association was unchanged in all comparison models. Further subgroup analyses stratified by the time of AD onset, and the quality of included studies provided statistical evidence of significant increased risk of AD in RR *versus* QQ+QR model only in late‐onset subjects (OR = 1.56, 95% CI = 1.07–2.26, *P* = 0.021) and in studies with high quality (OR = 1.37, 95% CI = 1.01–1.86, *P* = 0.043). This meta‐analysis suggests that the RR genotype in saitohin Q7R polymorphism may be a human‐specific risk factor for AD, especially among late‐onset AD subjects and caucasian populations.

## Introduction

AD, the most common type of dementia in ageing population, is characterized by progressive cognitive impairment and memory loss. Extracellular amyloid plaques and intracellular neurofibrillary tangles are two core pathological hallmarks of AD 1. Although the processes of AD could be triggered by many environmental factors, previous studies also suggested that genetic polymorphisms play an important role in AD, among which mutations in amyloid precursor protein (APP), presenilin‐1 (PSEN1), presenilin‐2 (PSEN2) and apolipoprotein E (APOE) have been proved to be associated with AD risk 2. However, AD is such a complex disorder that the genes mentioned above cannot explain the overall genetic susceptibility, and additional genetic risk factors may be involved in the development of AD.

STH, an intronless gene, was first discovered between exons 9 and 10 of the human microtubule‐associated protein tau (MAPT) gene on chromosome 17q21.1 and rediscovered in MAPT 5′ intron 11 3, 4. It encodes a 128‐amino acid protein with no clear homologues 3. This region is functionally critical for alternative splicing of exon 10, and the tissue expression of STH is similar to tau, which indicates STH gene a possible role in AD and other neurodegenerative disorders. A single‐nucleotide polymorphism [A→G] (rs62063857) in human STH gene results in an amino acid change from glutamine (Q) residue 7 to arginine (R). Conrad *et al*. (2002) first reported that the RR genotype and R allele were associated with a higher risk for late‐onset Alzheimer's disease (LOAD) independently from APOE‐4 genotype [odds ratio (OR), 11.92 for genotype; 3.11 for allele] 3. If this initial report was convincing, the Q7R polymorphism would become the second most important genetic susceptibility factor for AD. Subsequently, a large amount of studies were performed to confirm the important finding, whereas results were conflicting.

The issue has been discussed in one meta‐analysis published in 2004 5. However, the meta‐analysis failed to include all eligible studies–a study by Pepłon′ska *et al*., Oliveira *et al.,* and Clark *et al.,* in 2003 6, 7, 8. Additionally, a further eight papers focusing on the relationship between the Q7R polymorphism and AD susceptibility have emerged with inconsistent findings as the meta‐analysis was conducted 9, 10, 11, 12, 13, 14, 15, 16. Therefore, we performed this meta‐analysis of the existing studies to determine whether there is an association between STH Q7R polymorphism and AD risk.

## Materials and methods

### Literature search

This meta‐analysis was performed according to the methodology advocated by the Meta‐analysis of Observational Studies in Epidemiology (MOOSE) guideline 17. To identify all publications relevant to the association between STH Q7R polymorphism and AD, two investigators independently performed a systematic electronic literature search in the PubMed, Embase, Cochrane Library and Web of Science with the following terms: (‘Alzheimer's disease’ or ‘AD’) and (‘saitohin’ or ‘STH’) and (‘polymorphism’ or ‘mutation’ or ‘variant’). We also searched for additional publications in personal reference lists from original research articles and review articles. The articles selected were restricted to studies in humans and written in English, but without restriction on time period, sample size or population of the published paper. The last literature search was updated to 31 August 2016.

### Literature inclusion

All studies eligible for the meta‐analysis had to meet the following inclusion criteria: (*i*) studies designed as case–control type; (*ii*) writing in English; (*iii*) reporting the association between STH Q7R polymorphism and AD and (*iv*) providing detailed genotype counts essential for the calculation of ORs and 95% confidence intervals (CIs). Exclusion criteria were as follows: (*i*) study design based on family or sibling pairs; (*ii*) case reports, editorials, reviews and meta‐analyses and (*iii*) insufficient information for data extraction. Additionally, if there was more than one publication from the same population, only the most recent or comprehensive study was included in the meta‐analysis.

### Data extraction

The following information was extracted and tabulated by two independent reviewers: the first author's name, year of publication, country of origin, ethnicity, total number of cases and controls, mean age of cases and controls, gender proportion of cases and controls, diagnostic criteria of AD, time of AD onset, genotype and allele distributions of cases and controls, and *P* value for the control in the Hardy–Weinberg equilibrium (HWE). With regard to different results, a third reviewer participated in the discussion to solve the discrepancies.

### Quality assessment

The quality of the studies included in the meta‐analysis was evaluated by a set of predetermined criteria by Thakkinstian *et al*. (2005), which contains the representativeness of cases, representativeness of controls, ascertainment of cases, control selection, genotyping examination, HWE in controls and total sample size 18. The criteria have been previously structured as a 22‐item list with scores ranging from 0 to 15 and widely used in various meta‐analyses 19, 20. As in previous meta‐analyses, a study score ≥10 was considered to be high quality, while score <10 was considered to be low quality.

### Statistical analysis

Pooled ORs and 95% CIs were calculated to assess the association between STH Q7R polymorphism and the risk of AD under different comparison models, including allele model (R *versus* Q), dominant model (RR+QR *versus* QQ), recessive model (RR *versus* QQ+QR), homozygous model (RR *versus* QQ) and heterozygous model (QR *versus* QQ). Subgroup analyses were also performed to evaluate the effect of Q7R polymorphism on AD susceptibility according to the differences in time of AD onset (EOAD or LOAD) and quality score of included articles (high quality or low quality), respectively. Statistical heterogeneity between studies was tested by *I* ^2^ statistics and Q test. With the absence of heterogeneity (*I* ^2 ^< 50% or *P*_Q_ **≥ 0.1), the pooled ORs were merged by the fixed‐effects model (the Mantel–Haenszel method), while with the presence of heterogeneity (*I* ^2 ^≥ 50% or *P*_Q_ < 0.1), the random‐effects model (the Der Simonian and Laird method) was used. If heterogeneity was detected, Galbraith plot analyses were conducted to find out whether there were outliers that could be the potential sources of heterogeneity. The HWE was assessed by chi‐squared test using genotype data from controls. A sensitivity analysis for the overall effect was conducted by sequential removal of the four studies in which the HWE in the control group was not reported, as they may generate possible bias 9, 12, 14, 15. Publication bias was investigated with funnel plot and Egger's test (*P *< 0.05 indicated a significant publication bias). All statistical analyses were performed with STATA Version 12.0 (College Station, TX, USA).

## Results

### Study characteristics

**Figure 1:**
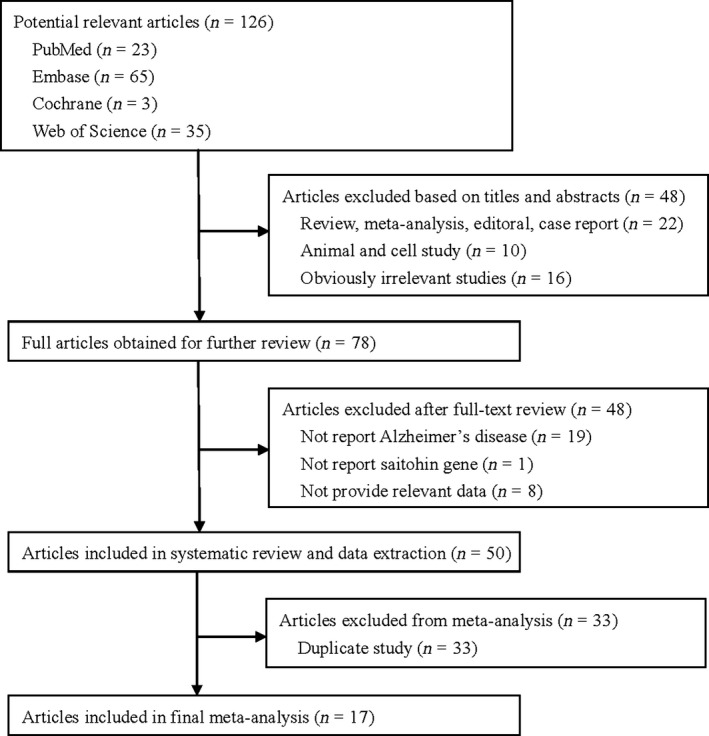
Flowchart of literature search.

A total of 126 articles were identified in the literature search of PubMed, Embase, Cochrane Library and Web of Science using different combinations of keywords (Fig. 1). After a careful review, 19 case–control studies from 17 articles with 4387 cases and 3972 controls were included in our meta‐analysis to determine the association between STH Q7R polymorphism and AD 3, 5, 6, 7, 8, 9, 10, 11, 12, 13, 14, 15, 16, 21, 22, 23, 24 (Table 1). Of the selected studies, 15 studies included populations of caucasian 3, 5, 6, 7, 8, 10, 11, 12, 13, 16, 21, 22, 23, 24, three of Asian 9, 14, 15 and one of African 8. The diagnostic criteria of AD for 10 studies were the National Institute of Neurological Disorders and Stroke/Alzheimer Diseases and Related Disorders Association criteria (NINCDS/ADRDA criteria) 6, 9, 10, 12, 13, 14, 16, 21, 23, 24, four studies were NINCDS/ADRDA criteria accompanied by the Consortium to Establish a Registry for Alzheimer's Disease (CERAD) or the third/fourth Diagnostic and Statistical Manual of Mental Disorders criteria (DSM‐III‐R/DSM‐IV criteria) 8, 11, 15, four studies were autopsy confirmed 3, 5, 10, 22 and one study was CERAD and National Institute on Aging and the Reagan Institute (NIA‐Reagan) criteria 7. Six studies only included late‐onset AD subjects 8, 13, 14, 15, 22, and the others included both early‐onset and late‐onset subjects 3, 5, 6, 7, 9, 10, 11, 12, 16, 21, 23, 24, among which AD in three studies was stratified into two age groups 6, 21, 24. All of the studies included met the quality criteria with scores ranging from 5 to 12; six studies were considered as high quality 7, 10, 13, 22, 23, 24, and 13 were low quality 3, 5, 6, 8, 9, 10, 11, 12, 14, 15, 16, 21. The genotype distributions of the controls in 15 studies were consistent with HWE (*P *> 0.05) 3, 5, 8, 10, 11, 13, 16, 21, 22, 23, 24, and the others were not reported 9, 12, 14, 15. Detailed characteristics of the studies included in this meta‐analysis are presented in Table 1. The distributions of genotypes and alleles in individual study are shown in Table 2.

**Table 1:** Study characteristics from included studies in the meta‐analysis

First author	Year	Country	Ethnicity	Case				Control			Diagnostic criteria of AD	Time of AD onset	QS
				*N* a	Age^b^	Age^c^	Gender^d^	*N*	Age^b^	Gender^d^			
Conrad	2002	USA	Caucasian	51	80.51	–	–	30	78.83	–	Autopsy‐confirmed	Mixed	7
Verpillat	2002	France	Caucasian	499	–	63.8	38%	402	66.6	48%	NINCDS‐ADRDA	Mixed	9
Cook	2002	British	Caucasian	203	81.4	>65	34.5%	309	82.1	41.1%	Autopsy‐confirmed	LOAD	12
Streffer	2003	Swiss, Greek	Caucasian	225	–	71.61	–	144	70.0	–	NINCDS‐ADRDA	Mixed	11
Pepłon′ska	2003	Polish	Caucasian	100	76.4	71.5	36%	100	71.2	21%	NINCDS‐ADRDA	Mixed	9
Oliveira	2003	USA	Caucasian	903	–	–	–	320	–	–	CERAD NIA‐Reagan	Mixed	11
Clark‐1	2003	Whites, Hispanics	Caucasian	135	81.6	–	22.5	340	75.7	35.8	NINCDS‐ADRDA DSM‐III‐R	LOAD	9
Clark‐2	2003	African‐Americans	African	65				118			NINCDS‐ADRDA DSM‐III‐R	LOAD	9
Combarros	2003	Spain	Caucasian	315	75.6	71.9	30%	307	80.5	28%	NINCDS‐ADRDA	Mixed	10
Tanahashi	2004	Japanese	Asian	15	–	–	–	15	–	–	NINCDS‐ADRDA	Mixed	6
Seripa‐1	2004	USA	Caucasian	117	80.89	71.65	45.3%	99	83.75	46.5%	Autopsy‐confirmed (P)	Mixed	10
Seripa‐2	2004	Italian	Caucasian	130	69.80	65.91	42.3%	633	36.76	48.2%	NINCDS‐ADRDA	Mixed	9
Conrad	2004	Germany	Caucasian	155	80.9	–	–	41	76.1	–	Autopsy‐confirmed	Mixed	7
Johansson	2005	Sweden	Caucasian	398	77	73	41.7%	186	72	43.5%	NINCDS‐ADRDA CERAD	Mixed	9
Zuo	2006	European‐Americans	Caucasian	286	–	–	36.7%	197	–	44.2%	NINCDS‐ADRDA	Mixed	8
Mateo	2006	Spain	Caucasian	139	75.7	72.1	33%	235	80.4	30%	NINCDS‐ADRDA	LOAD	11
Wang	2008	Chinese	Asian	207	77.6	–	48%	222	72.3	61%	NINCDS‐ADRDA	LOAD	5
Lin	2008	Chinese	Asian	280	79.72	75.75	24.6%	220	78.49	28.2%	NINCDS‐ADRDA DSM‐IV	LOAD	8
Lorenzi	2010	Italian	Caucasian	164	75.49	71.63	28.7%	54	66.79	51.9%	NINCDS‐ADRDA	Mixed	7

USA: United states of America; NINCDS: the National Institute of Neurological Disorders and Stoke; ADRDA: Alzheimer Diseases and Related Disorders Association; CERAD: the Consortium to Establish a Registry for Alzheimer's Disease; NIA‐Reagan: the National Institute on Aging and the Reagan Institute; DSM: the Diagnostic and Statistical Manual of Mental Disorders; AD: Alzheimer's disease; LOAD: late‐onset Alzheimer's disease; QS: quality score.

a Number.

b Age at examination.

c Age at onset of Alzheimer's disease.

d Percentage of male.

**Table 2:** Genotype and allele distribution of saitohin Q7R polymorphism among AD cases and controls in the included studies

First author, year	Cases					Controls					HWE^a^
	QQ	QR	RR	Q	R	QQ	QR	RR	Q	R	
Conrad, 2002	26	17	8	69	33	22	8	0	52	8	0.399
Verpillat, 2002	272	189	38	733	265	222	161	19	605	199	0.132
Cook, 2002	119	73	11	311	95	190	104	15	484	134	0.874
Streffer, 2003	144	68	13	356	94	84	56	4	224	64	0.134
Pepłon′ska, 2003	76	23	1	175	25	74	23	3	171	29	0.469
Oliveira, 2003	570	287	46	1427	379	189	110	21	488	152	0.362
Clark‐1, 2003	97	33	5	227	43	226	97	17	549	131	0.127
Clark‐2, 2003	57	8	0	122	8	106	12	0	224	12	0.561
Combarros, 2003	177	109	29	463	167	170	120	17	460	154	0.482
Tanahashi, 2004	15	0	0	30	0	15	0	0	30	0	NR
Seripa‐1, 2004	70	41	6	181	53	71	26	2	168	30	0.831
Seripa‐2, 2004	66	56	8	188	72	357	230	46	944	322	0.290
Conrad, 2004	111	36	8	258	52	24	14	3	62	20	0.635
Johansson, 2005	303	89	6	695	101	132	50	4	314	58	0.771
Zuo, 2006	–	–	–	439	133	–	–	–	313	81	NR
Mateo, 2006	121		18	–	–	221		14	–		0.79
Wang, 2008	207	0	0	414	0	222	0	0	444	0	NR
Lin, 2008	279	1	0	559	0	220	0	0	440	0	NR
Lorenzi, 2010	105	59		–	–	28	26		–	–	>0.05

AD: Alzheimer's disease; HWE: Hardy–Weinberg equilibrium; NR: not reported.

a *P* value for HWE test in controls.

### Quantitative synthesis

**Figure 2:**
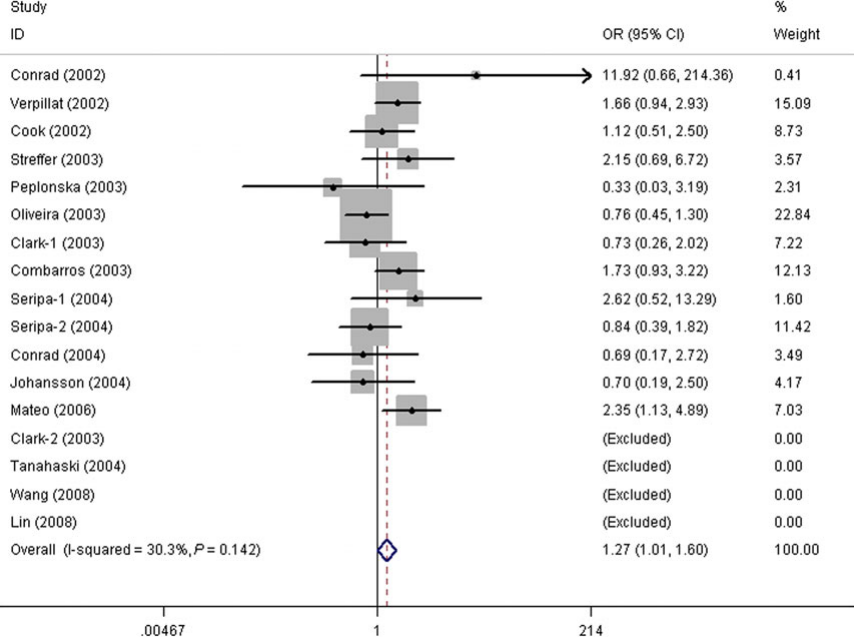
Forest plots of saitohin Q7R polymorphisms and Alzheimer's disease's risk in RR *versus* QQ+QR model (fixed‐effects model). (OR = 1.27, 95% CI = 1.01–1.60, *P* = 0.040).

The results of the overall meta‐analysis suggested that the Q7R polymorphism was significantly associated with an increased risk of AD in recessive model (RR *versus* QQ+QR, OR = 1.27, 95% CI = 1.01–1.60, *P* = 0.040; Fig. 2). Due to the limited number of studies, we could not stratify by ethnicity (three studies in Asians and one in African) 8, 9, 14, 15, but after excluding the four studies not carried out in caucasians, the overall association was unchanged in all comparison models (Table 3). When stratified by the time of AD onset, we found the association between Q7R polymorphism and AD susceptibility was only significant in LOAD in RR *versus* QQ+QR model (OR = 1.56, 95% CI = 1.07–2.26, *P* = 0.021; Table 3). A similar situation was also found in subgroup analysis stratified by the quality of included studies, where in the recessive model, the Q7R polymorphism was significantly related to AD risk only in studies with high quality (OR = 1.37, 95% CI = 1.01–1.86, *P* = 0.043), while a null result was reported in studies with low quality in all genetic models (Table 3).

**Table 3:** Meta‐analysis and heterogeneity test of the saitohin Q7R polymorphism and Alzheimer's disease

Subgroups	Allele model (R *versus* Q)				Dominant model (RR+QR *versus* QQ)				Recessive model (RR *versus* QQ+QR)				Homozygous model (RR *versus* QQ)				Heterozygous model (QR *versus* QQ)			
	OR(95% CI)	*P*	*I* ^2^	*P*_Q_	OR(95% CI)	*P*	*I* ^2^	*P*_Q_	OR(95% CI)	*P*	*I* ^2^	*P*_Q_	OR(95% CI)	*P*	*I* ^2^	*P*_Q_	OR(95% CI)	*P*	*I* ^2^	*P*_Q_
Total	1.02(0.90–1.15)	0.761	37.7	0.069	0.95(0.85–1.06)	0.390	29.2	0.137	**1.27(1.01**–**1.60)**	**0.040**	30.3	0.142	1.17(0.92–1.50)	0.206	24.3	0.205	0.94(0.84–1.06)	0.330	9.4	0.349
Caucasian	1.02(0.90–1.15)	0.814	45.5	0.037	0.95(0.82–1.10)	0.475	37.4	0.085	**1.27(1.01**–**1.60)**	**0.040**	30.3	0.142	1.17(0.92–1.50)	0.206	24.3	0.205	0.94(0.84–1.06)	0.290	19.8	0.250
Time of AD onset																				
EOAD	1.00(0.81–1.23)	0.985	0.0	0.812	0.91(0.71–1.18)	0.478	0.0	0.974	1.47(0.86–2.52)	0.164	0.0	0.517	1.37(0.79–2.38)	0.263	0.0	0.545	0.86(0.66–1.12)	0.254	0.0	0.960
LOAD	1.09(0.92–1.29)	0.315	0.0	0.663	1.07(0.87–1.31)	0.543	0.0	0.759	**1.56(1.07–2.26)**	**0.021**	19.6	0.290	1.34(0.85–2.09)	0.205	10.5	0.340	1.03(0.83–1.23)	0.779	0.0	0.795
Quality score																				
High	1.01(0.88–1.14)	0.933	42.0	0.142	0.96(0.82–1.12)	0.597	37.3	0.172	**1.37(1.01–1.86)**	**0.043**	42.4	0.123	1.19(0.85–1.67)	0.317	34.0	0.194	0.93(0.79–1.10)	0.385	33.5	0.198
Low	1.01(0.84–1.21)	0.939	42.1	0.077	0.95(0.81–1.10)	0.493	32.8	0.146	1.15(0.81–1.64)	0.433	23.5	0.250	1.15(0.80–1.66)	0.436	28.7	0.209	0.96(0.81–1.13)	0.612	3.4	0.407

AD: Alzheimer's disease; EOAD: early‐onset Alzheimer's disease; LOAD: late‐onset Alzheimer's disease; OR: odds ratio; CI: confidence intervals.

### Heterogeneity analysis

**Figure 3:**
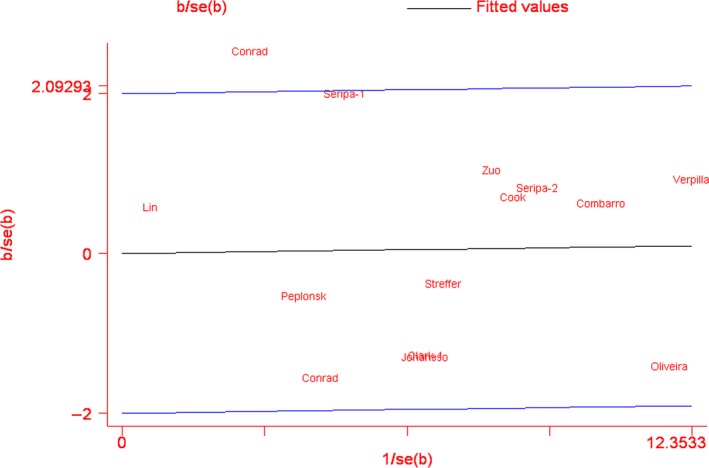
Galbraith plot of Saitohin Q7R polymorphism and Alzheimer's disease risk. The study by Conrad et al was the outlier in R *versus* Q model in the overall analysis.

For Q7R polymorphism, there was heterogeneity in R *versus* Q model when all eligible studies were included into meta‐analysis (*I* ^2^ = 37.7%, *P*_Q_ = 0.069; Table 3). Galbraith plot analysis indicated that Conrad *et al*. (2002) was the outlier and main contributor to heterogeneity in R *versus* Q model (Fig. 3) 3. When omitting the outlier study, the insignificance of the OR was not altered but heterogeneity decreased (*I* ^2^ = 17.4%, *P*_Q_ = 0.264). After excluding the four studies not conducted in caucasians 8, 9, 14, 15, between‐study heterogeneities were detected under R *versus* Q model (*I* ^2^ = 45.5%, *P*_Q_ = 0.037) and RR+QR *versus* QQ model (*I* ^2^ = 37.4%, *P*_Q_ = 0.085; Table 3). After stratifying by the time of AD onset, the heterogeneity disappeared [*I* ^2^ = 0.0% for all genetic models for both EOAD and LOAD, except for recessive model and homozygous model in LOAD (*I* ^2^ = 19.6%, *P*_Q_ = 0.290; *I* ^2^ = 10.5%, *P*_Q_ = 0.340, respectively)] (Table 3). When subgroup analyses were performed in all comparison models, obvious significant heterogeneities were still observed in studies with low quality after stratified according to the quality score of included studies (R *versus* Q: *I* ^2^ = 42.1%, *P*_Q_ = 0.077; Table 3).

### Sensitivity analysis and bias diagnosis

**Figure 4:**
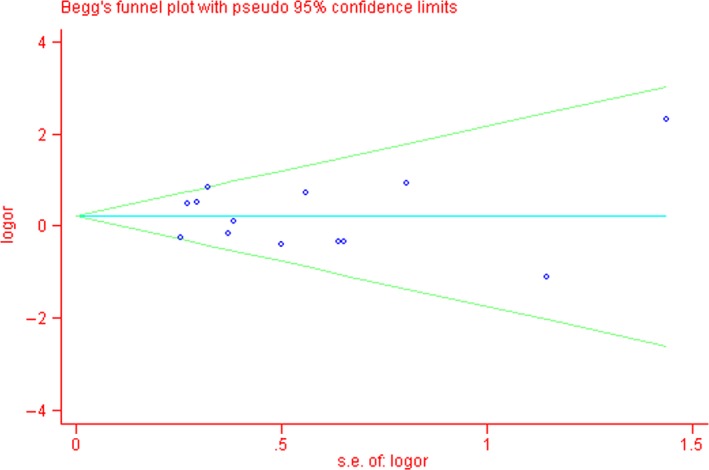
Funnel plot analysis and Egger's test of Q7R polymorphism and Alzheimer's disease risk. Each point represents a separate study for the indicated association. Funnel plot for contrast RR *versus* QQ+QR in the overall analysis (*P* = 0.984).

As the HWE of the control group in four studies was not reported, sensitivity analyses were performed by omitting one study each time 9, 12, 14, 15. The significances of ORs were not changed through the exclusion of any single study in all comparison models (data not shown). Funnel plot and Egger's test were conducted to assess possible publication bias. Ultimately, both funnel plot and Egger's test indicated no evidence of publication bias (Fig. 4).

## Discussion

AD is a complex disorder with multiple genetic and environmental factors that may have influences on disease susceptibility. However, the aetiology and pathogenesis of AD are not fully understood. To date, many researchers have reported the association of AD with gene polymorphism, among which APP, PSEN1, PSEN2 and APOE gene are widely accepted as important risk factors in AD. The association between STH Q7R polymorphism and AD has been investigated for many years, but the results remain controversial. As the single studies may have inadequate statistical power, here we performed a meta‐analysis known as an important tool to precisely evaluate the relationship between Q7R polymorphism and the risk of AD. We included 4387 cases and 3972 controls in this article. Meta‐analysis showed that the RR genotype of STH Q7R polymorphism was associated with an increased risk for AD. Subgroup analysis indicated that RR genotype of STH Q7R polymorphism leads to the increased risk of LOAD, but not EOAD. When stratified by the quality score of included studies, the RR genotype was found contributing to the increased risk of AD only in high‐quality studies.

The special localization of STH gene in a functionally critical position of the tau gene could explain its role in tauopathies. As STH locates in the intron between exons 9 and 10 of tau, there is a possibility that STH Q7R polymorphism may mediate the different expressions of tau isoforms through influencing alternative splicing of exon 10 25. Alternative splicing of exon 10 defines two functionally different isoforms with either four repeats (4R) or three repeats (3R) depending on whether exon 10 is included or not 26. In normal adult human brains, the level of 3R isoforms is approximately equal to that of 4R isoforms 27. It was shown that 4R tau has a threefold binding affinity to tubulin than 3R tau and assembles microtubules more effectively as compared to 3R tau. The 4R‐to‐3R ratio appears to be essential for preventing neurodegeneration. Additionally, the Q7R polymorphism was in complete linkage disequilibrium with two extended tau haplotypes: The Q allele is located on the H1 tau haplotype, and the R allele is located on the H2 tau haplotype, respectively 21. With comparison to H1 tau haplotype carriers among frontotemporal lobar degeneration (FTLD) patients, H2 tau haplotype carriers had hypoperfusion of frontal medial and cingulated cortex 28 and higher cerebrospinal fluid total tau and phospho tau 29. Evidence from population‐based studies also showed that the H2 MAPT haplotype was associated with FTLD and AD 30, 31, 32.

Between‐study heterogeneity is very common in meta‐analyses for genetic association studies, and it is necessary to find out the potential sources. Our meta‐analysis also showed significant heterogeneity in allele model in the overall effects. Galbraith plot analysis indicated that Conrad *et al*.'s (2002) study was the outlier. Conrad *et al*.'s (2002) study was first to report the association between Q7R polymorphism and AD risk with 51 cases and 30 controls. Due to the small number of subjects, result from this study was not convincing and may have potential bias. Furthermore, subgroup analyses were performed to explore the sources of heterogeneity and the stability of the result. Age is a very important factor for AD development, and most of AD is diagnosed in people over 65 years. Results from the stratification by the time of AD onset showed that AD risk was associated with late onset in recessive model, which indicated that the STH Q7R polymorphism may be age‐dependently associated with AD susceptibility. Possible explanation for the age‐dependent association could be the difference in circulating C‐reactive protein (CRP) level, a well‐known inflammatory biomarker involved in the pathogenesis of AD 33, 34. Previous research demonstrated that CRP level was significantly higher in LOAD than EOAD 35. When stratified by the quality score of included study, between‐study heterogeneity was found only in studies with low quality, suggesting that the differences of individual study's quality may be the potential confounder. Moreover, AD is such a multi‐factorial disease in relation to many gene variants and environmental factors that other genetic and environmental variables, as well as their possible interaction, may be potential contributors.

The association of STH gene polymorphism with AD risk has been evaluated by a previous meta‐analysis with six studies included 5. The results suggested that the RR genotype had a highly significant trend towards overrepresentation in AD compared with normal control subjects; however, the R allele was not significantly overexpressed in AD subjects. What's more, the meta‐analysis failed to include all eligible studies, and heterogeneity test and sensitivity analysis were not applied to this. In the present meta‐analysis of data from 19 case–control studies, we also found a notable increase in risk of AD and Q7R polymorphism. Additionally, we noticed this association only significantly existed in LOAD subjects, as well as in studies with high quality, whereas the small number of included studies in the earlier work limited the stratification.

To our knowledge, STH is an evolutionary locus that separates humans and their closest relatives from other mammals. The Q allele is remarkably common in humans; however, all nonhuman primates are homozygous for the R allele, which makes the Q allele a human‐specific marker and can be inferred to be most implicated in Alzheimer pathogenesis 36. Nevertheless, similar to previous meta‐analyses, there were also several limitations in the current study. First, the sample size of most eligible studies is relatively small and we had no ability to confirm whether studies included in our meta‐analysis had sufficient genetic power. Meanwhile, the results are not currently available from the Alzheimer Genome sequencing project, which includes more than 5000 patients and controls to strengthen the population genotype statistics. Second, we only included studies in English and might lead to language bias. According to Pan *et al*. (2005), the influence of language bias on meta‐analyses of observational studies may be as large as or even larger than its influence on randomized evidence 37. Third, the overall results of our study were derived from crude ORs due to lack of the original data, such as age, gender, ethnicity, education level and APOE ε4 status. Fourth, publication bias may exist because of no attempt to obtain unpublished studies, although both funnel plot and Egger's test indicated no evidence. In addition, current limited knowledge of STH structure–function relationships and clinical features are non‐negligible issues that do unfortunately weaken our results.

In conclusion, our meta‐analysis suggests that the RR genotype of STH Q7R polymorphism may be associated with an increased risk for AD, especially in caucasian population, late‐onset AD subjects and studies with high quality. Considering the limitations mentioned above, further well‐designed epidemiological studies with larger sample size and structure–function relationships should be conducted to confirm our findings.

## Conflict of interest

The authors declare no conflict of interest.

## Author contribution

Shaohua Wang and Rong Huang contributed to study conception and design; Rong Huang and Sai Tian acquired the data; Rongrong Cai, Jie Sun, Wenqing Xia, Xue Dong and Yanjue Shen performed the analyses; Rong Huang wrote the first draft; and Shaohua Wang and Sai Tian revised it critically for important intellectual content. All authors approved the final version to be published.
